# Utility of three cognitive screening tests to triage referrals to a dementia specialty clinic

**DOI:** 10.1002/alz.089754

**Published:** 2025-01-03

**Authors:** Robin C. Hilsabeck, Jared F Benge, Bonnie M Scott, Soumya Thotakura, Amtul‐Noor Rana, Suzanne Schmitz

**Affiliations:** ^1^ UT Health San Antonio, San Antonio, TX USA; ^2^ University of Texas–Austin, Austin, TX USA; ^3^ The University of Texas at Austin Dell Medical School, Austin, TX USA; ^4^ University of Texas at Austin, Austin, TX USA; ^5^ Dell Medical School at The University of Texas at Austin, Austin, TX USA

## Abstract

**Background:**

Due to the shortage of healthcare professionals with expertise in diagnosis and treatment of Alzheimer disease and related dementias, there are long wait times to be evaluated in dementia specialty clinics and no clear guidance about how to allocate limited resources. The purpose of this study was to examine utility of cognitive screening measures administered by clinic staff to determine level of cognitive impairment to aid in decisions about which patients may benefit from full diagnostic services.

**Methods:**

Participants were 169 older adults who completed an intake interview, including a brief cognitive screening test, conducted by a neuropsychologist at a dementia specialty clinic. During the rooming process, clinic staff administered one of three cognitive screening measures, Quick Dementia Rating Scale (QDRS; n = 47), Quick Mild Cognitive Impairment (Qmci) screen (n = 70), and the BrainCheck Standard Battery (n = 52). After the intake interview, blinded to results of these cognitive screening measures, the neuropsychologist completed an initial impressions form indicating level of cognitive impairment (i.e., none, mild, possible dementia, definite dementia). A separate neuropsychologist, blinded to information from the intake interview, completed the same form based on results of each of the three cognitive screening measures and patient demographic information.

**Results:**

Based on impressions from the intake interview, 27 patients were cognitively normal (CN), 67 had mild cognitive impairment (MCI), 32 had possible dementia, and 43 had definite dementia. Agreement on level of cognitive impairment between the intake interview and cognitive screening measures was 53% for QDRS (κ = .459, p < .001), 41% for Qmci (κ = .423, p < .001), and 48% for BrainCheck (κ = .539, p < .001). Agreement improved notably for all three measures when level of cognitive impairment was limited to two groups (i.e., CN + MCI vs possible + definite dementia): 79% for QDRS (κ = .566), 77% for Qmci (κ = .537), and 83% for BrainCheck (κ = .655).

**Conclusions:**

Cognitive screening measures administered by clinic staff and interpreted by dementia experts may have utility as time‐efficient, cost‐effective clinical decision support tools for the allocation of limited dementia diagnostic resources.

